# Betulin Promotes Differentiation of Human Osteoblasts In Vitro and Exerts an Osteoinductive Effect on the hFOB 1.19 Cell Line Through Activation of JNK, ERK1/2, and mTOR Kinases

**DOI:** 10.3390/molecules24142637

**Published:** 2019-07-19

**Authors:** Magdalena Mizerska-Kowalska, Adrianna Sławińska-Brych, Katarzyna Kaławaj, Aleksandra Żurek, Beata Pawińska, Wojciech Rzeski, Barbara Zdzisińska

**Affiliations:** 1 Department of Virology and Immunology; Maria Curie-Sklodowska University, Lublin 20-033, Poland; 2 Department of Cell Biology, Maria Curie-Sklodowska University, Lublin 20-033, Poland; 3 Department of Medical Biology, Institute of Rural Health, Lublin 20-090, Poland

**Keywords:** betulin, osteoblasts, differentiation, mineralization

## Abstract

Although betulin (BET), a naturally occurring pentacyclic triterpene, has a variety of biological activities, its osteogenic potential has not been investigated so far. The aim of this study was to assess the effect of BET on differentiation of human osteoblasts (hFOB 1.19 and Saos-2 cells) in vitro in osteogenic (with ascorbic acid as an osteogenic supplement) and osteoinductive (without an additional osteogenic supplement) conditions. Osteoblast differentiation was evaluated based on the mRNA expression (RT-qPCR) of Runt-related transcription factor 2 (RUNX2), alkaline phosphatase (ALP), type I collagen-α1 (COL1A1), and osteopontin (OPN). Additionally, ALP activity and production of COL1A1 (western blot analysis) and OPN (ELISA) were evaluated. The level of mineralization (calcium accumulation) was determined with Alizarin red S staining. BET upregulated the mRNA level of RUNX2 and the expression of other osteoblast differentiation markers in both cell lines (except the influence of BET on ALP expression/activity in the Saos-2 cells). Moreover, it increased mineralization in both cell lines in the osteogenic conditions. BET also increased the mRNA level of osteoblast differentiation markers in both cell lines (except for ALP in the Saos-2 cells) in the osteoinductive conditions, which was accompanied with increased matrix mineralization. The osteoinductive activity of BET in the hFOB 1.19 cells was probably mediated via activation of MAPKs (JNK and ERK1/2) and mTOR, as the specific inhibitors of these kinases abolished the BET-induced osteoblast differentiation. Our results suggest that BET has the potential to enhance osteogenesis.

## 1. Introduction

Bone is an extremely heterogeneous tissue built up of cells and mineralized extracellular matrix [1]. It undergoes an unceasing process of remodeling that involves removal of old bone by osteoclasts and formation of new bone by osteoblasts. In physiological conditions, these events are balanced and provide tissue homeostasis [2]. However, in some cases, an imbalance in bone remodeling may lead to the development of bone diseases, e.g., osteopenia and osteoporosis. Osteoporosis is mainly associated with aging. Moreover, various diseases including rheumatoid arthritis, chronic inflammatory periodontal diseases, multiple myeloma, bone metastatic malignant cancers, or drugs adversely affect bone health and can contribute to the development of osteoporosis. Therefore, osteoporosis is considered as a global public health problem [3,4]. Currently, therapeutic options employed for managing of osteoporosis include antiresorptive agents whose actions is directed mainly to osteoclasts and bone anabolic drugs that regulate the function of osteoblasts. However, both types of therapies may have adverse side effects, especially upon long-term use in the elderly or patients having multimorbidity [5,6]. Therefore, new anti-osteoporotic agents are still being sought.

Differentiation of osteoblasts from mesenchymal stem cells to mature osteoblasts depositing mineralized matrix is regulated by extracellular signals that activate intracellular signaling pathways and regulate the action of several osteogenesis-related transcription factors, including Runt-related transcription factor 2 (RUNX2) [7]. RUNX2 is considered to be the master transcription factor, as it regulates the expression of genes linked with osteoblast maturation such as *ALPL* (alkaline phosphatase, ALP), *Col1A1* (collagen α1 type I, COL1), *SPP1* (osteopontin, OPN), *IBSP* (bone sialoprotein II, BSPII), and *BGLAP* (osteocalcin, OCN) [7]. Different signaling systems regulate bone formation, but mitogen-activated kinase (MAPK) and mammalian target of rapamycin (mTOR) pathways play a key role in this process, as they affect osteoblast differentiation [8,9]. MAP kinases, i.e., extracellular signal-regulated kinases (ERK1/2) and p38, have been identified as regulators of RUNX2 activation [10,11], while c-Jun N-terminal protein kinases (JNKs) regulate the expression of activating transcription factor 4 (ATF4) and are required for late-stage osteoblast differentiation [12]. Also, both mTOR complexes, i.e., mTORC1 and mTORC2, are involved in osteoblast differentiation [9]. More recently, it has been shown that mTORC1 promotes osteoblast differentiation through the regulation of RUNX2 expression [13].

At present, medicines or supplements derived from natural sources have aroused wide interest. Plant extracts are especially rich in diverse active compounds. One of them are pentacyclic triterpenes with a lupane skeleton, to which betulin (BET; lup-20(29)-ene-3β,28-diol) is included [14,15]. BET is found predominantly in the bark of trees of the genus Betula (Betulaceae), which are well known as a rich source of compounds with healing properties [15]. This triterpene exhibits a wide range of pharmacological effects [14], including anticancer [16,17,18], anti-viral [19,20], and anti-pathogenic [21] activities. Due to its anti-inflammatory and anti-oxidative activities, betulin may also exert hepato- or cardioprotective properties [22,23,24,25]. Moreover, BET exhibits analgesic [26] and anti-hyperlipidemic [27] activities. Betula bark and bark extracts have been known in traditional medicine and have been used for treatment of various diseases, including micro-fracture and dislocated bone [15]. Recently, it has been shown that pentacyclic triterpenoids such as ursolic, corosolic, and betulinic acid can influence bone formation as they enhance osteoblast differentiation [28,29,30,31]. However, to the best of our knowledge, the effect of betulin on osteogenesis has never been studied before. These all data prompted us to evaluate whether BET exerts anabolic activity by engagement in bone formation. To this end, we examined the effects of betulin on the differentiation and mineralization of osteoblasts of two human cell lines both in the presence of an osteogenic medium and without an osteogenic supplement such as ascorbic acid [32,33]. Moreover, some signaling mechanisms involved in the pro-osteogenic activity of BET were studied.

## 2. Results

### 2.1. Effect of BET on the Viability and Proliferation of Osteoblasts

**Figure 1:**
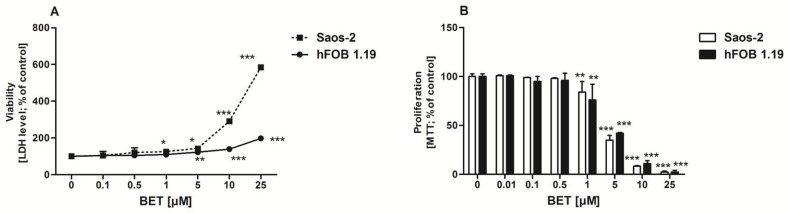
Effect of BET on hFOB 1.19 and Saos-2 cell viability (**A**) and proliferation (**B**). The osteoblasts were treated with the indicated concentrations of the compound and cell viability was estimated with the LDH assay after 24 h or cell proliferation was determined with the MTT method following the 96-h BET treatment. The results represent the mean ± SD of three independent experiments (*n* = 24 per concentration), * *p* < 0.05, ** *p* < 0.01, *** *p* < 0.001 in comparison to the control, one-way ANOVA with post hoc Dunnett’s test.

Initially, to avoid the cytotoxicity of the compound towards osteoblasts, the effect of BET on the viability of hFOB 1.19 and Saos-2 cells was determined by the LDH assay. This test is one of the major methods for assessment of cell membrane integrity and, thus, the ability of the tested compound to disintegrate cells [34]. As shown in Figure 1A, BET decreased the viability of both osteoblast cell lines in a concentration-dependent manner. It was not toxic to the hFOB 1.19 cells up to 1 µM and to the Saos-2 cells up to 0.5 µM. Statistically significant LDH release appeared at 1 µM (Saos-2 cells) and 5 µM (hFOB 1.19 cells) of BET. The exposure of the osteoblasts to 25 µM of BET resulted in very high LDH leakage from the Saos-2 cells (more than six times higher than the control level), while only a minor cytotoxic effect was observed in the hFOB 1.19 cells. This revealed low toxicity of BET to the normal hFOB 1.19 osteoblasts, while the osteosarcoma Saos-2 cells were more sensitive to the compound.

As anabolic agents can increase bone formation either by increasing the proliferation and/or differentiation of osteoblast precursors [35], further investigation was undertaken to determine the effect of BET on osteoblast proliferation. As shown in Figure 1B, BET did not influence the proliferation of both cell lines up to 0.5 µM. However, at a concentration of 1 µM and higher doses, BET inhibited proliferation of osteoblasts in a concentration-dependent manner. BET used at the 25 µM concentration suppressed the proliferation of both osteoblast cell lines almost completely. Based on the viability/proliferation tests, only the non-toxic concentrations of BET, i.e., 0.01, 0.1, and 0.5 µM, were selected for further experiments.

### 2.2. BET Elevated the mRNA Level of RUNX2 and the Expression of other Osteoblast Differentiation Markers, and Enhanced the Level of Bone Matrix Mineralization in Osteogenic Conditions

**Figure 2:**
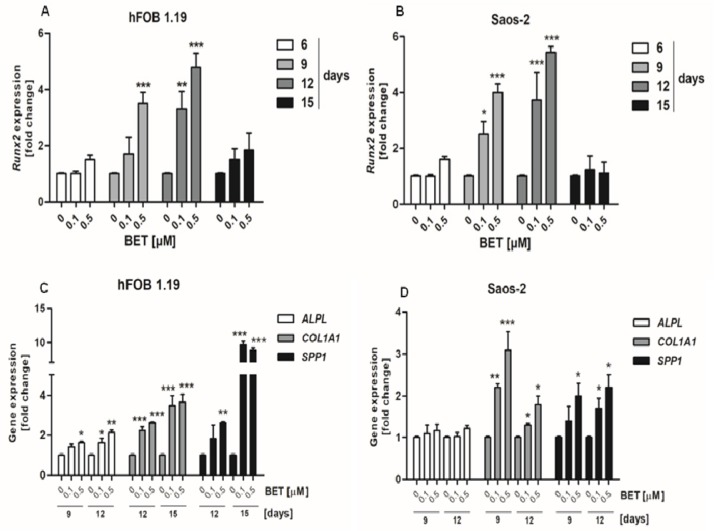
Effect of BET on the expression of *RUNX2* and other differentiation marker genes in osteoblasts in osteogenic conditions. The osteoblasts were cultured in an osteogenic medium supplemented with 0.1 or 0.5 μM of BET or without the compound (control). The expression of the *RUNX2* gene was determined in the hFOB 1.19 (**A**) and Saos-2 (**B**) cells by RT-qPCR after the indicated period of culture. The expression of osteogenic genes *ALPL*, *COLIA1* (type I collagen), and *SPP1* (OPN) was determined in the hFOB 1.19 (**C**) and Saos-2 (**D**) cells by RT-qPCR after the indicated period of culture. Mean ± SD from three independent experiments; * *p* < 0.05, ** *p* < 0.01, *** *p* < 0.001 in comparison to the control, one-way ANOVA with post hoc Dunnett’s test.

The osteogenic potential of different compounds in osteoblast cultures in vitro is usually tested in the presence of osteogenic supplements. Therefore, we evaluated the influence of BET on differentiation of osteoblasts in osteogenic conditions, namely in a medium supplemented with ascorbic acid [32,33]. First, we determined whether BET was able to modulate the expression of *RUNX2* gene, as the product of this gene, i.e., RUNX2 a transcription factor that regulates the degree of expression of other osteoblast differentiation markers in human osteoblasts [7]. The RT-qPCR assay revealed that BET used at low concentrations such as 0.1 and 0.5 µM was able to increase *RUNX2* expression in both osteoblast cell lines. The statistically significant enhancement of the RUNX2 mRNA level in comparison with the control was detected at day 9 and 12 of culture (Figure 2A,B). BET also enhanced the expression of other differentiation markers such as ALP (marker of the early phase of osteoblast differentiation), COL1A1 (marker of the late stage of osteoblast differentiation), and OPN (marker of the terminal stage of osteoblast differentiation). As shown in Figure 2C, the triterpenoid (0.1 and 0.5 µM) significantly enhanced the *ALPL*, *COL1A1*, and *SPP1* (OPN) gene expression in the hFOB 1.19 cells, in comparison with the control cells. BET influenced especially the expression of *COL1A1* and *SPP1* and, to a lesser extent, the expression of *ALPL* in the hFOB 1.19 cells (Figure 2C). However, in the Saos-2 cells, BET did not influence the expression of the *ALPL* gene, but it enhanced significantly the expression of both other markers (*COL1A1* and *SPP1*) in a concentration-dependent manner (Figure 2D).

**Figure 3:**
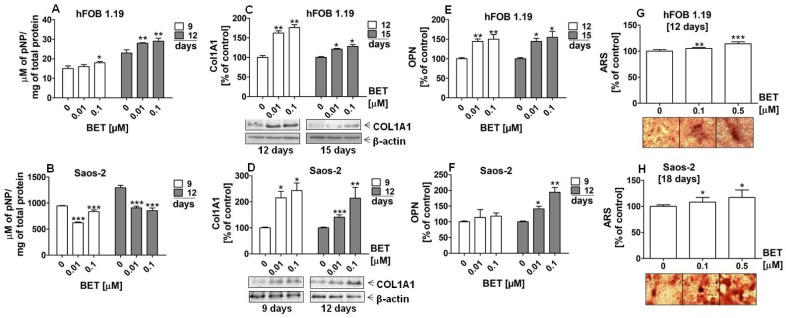
Effect of BET on the production of differentiation markers and the levels of mineralization in osteoblasts after the indicated period of culture in osteogenic conditions. The osteoblasts were cultured in an osteogenic medium supplemented with 0.01, 0.1, or 0.5 μM of BET or without the compound (control). ALP activity in the hFOB 1.19 (**A**) and Saos-2 cells (**B**) was measured with the colorimetric method. The levels of COL1A1 in the hFOB1.19 (**C**) and Saos-2 (**D**) cells were determined with the western blotting method. The levels of OPN in the culture media from the hFOB 1.19 (**E**) and Saos-2 (**F**) cells were evaluated with the ELISA method. The levels of calcium deposited in the extracellular matrix were determined in 24-well plates using Alizarin Red S staining. Representative micrographs (40×) of stained osteoblast cultures and mean levels of ARS extracted from the hFOB 1.19 (**G**) and Saos-2 cultures (**H**). Mean ± SD from three independent experiments; * *p* < 0.05, ** *p* < 0.01, *** *p* < 0.001 in comparison to the control, one-way ANOVA with post hoc Dunnett’s test.

Next, we evaluated the ALP activity and production of COL1A1 and OPN by osteoblasts to confirm additionally that BET promotes differentiation of osteoblasts. In this part of the study, BET was used in very low concentrations such as 0.01 and 0.1 µM. As shown in Figure 3A, the ALP activity in the hFOB 1.19 cell cultures started to increase at day 9 of incubation with 0.1 µM BET. Then, ALP reached maximal activity after treatment with 0.01 and 0.1 µM of BET at day 12 of culture. In contrast, a statistically significant decrease in the ALP activity was observed in the Saos-2 cell cultures at the same time points (Figure 3B). Moreover, western blot analysis revealed that BET enhanced the production of COL1A1 in the hFOB 1.19 and Saos-2 cells in a concentration-dependent manner, compared with the controls (Figure 3C,D). As shown in Figure 3E,F, the treatment of both cell lines with BET also enhanced the production of OPN after 12 days of incubation. In the case of the hFOB 1.19 cells, a similar effect of the triterpene treatment was also observed at day 15 of culture.

Finally, we conducted the ARS staining assay for qualitative and quantitative analysis of the mineralization process in the BET-treated hFOB 1.19 and Saos-2 cell cultures. The microscopic analysis of the ARS-stained osteoblast cultures revealed the appearance of evident nodules at day 12 of hFOB 1.19 and day 18 of Saos-2 differentiation (Figure 3G,H). Moreover, the ARS quantitative analysis indicated that calcium deposition was elevated by approx. 10% and 20% in the hFOB 1.19 cell cultures treated with 0.1 µM and 0.5 µM of BET, respectively, in comparison with the control. A similar increase in the level of mineralization was also observed in the Saos-2 cells at day 18 of the study.

### 2.3. BET Elevated the mRNA Level of Osteoblast Differentiation Markers and Enhanced the Level of Bone Matrix Mineralization in Osteoinductive Conditions

**Figure 4:**
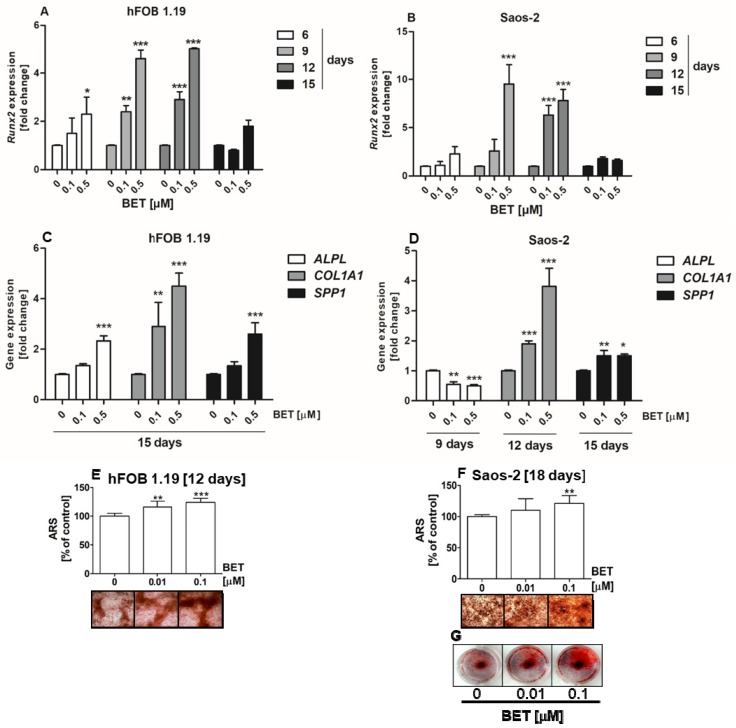
Effect of BET on the expression of *RUNX2* and other differentiation marker genes, and the levels of mineralization in osteoblasts in osteoinductive conditions. The osteoblasts were cultured in an osteoinductive medium supplemented with 0.01, 0.1, or 0.5 μM of BET or without the compound (control). The expression of the *RUNX2* gene was determined in the hFOB 1.19 (**A**) and Saos-2 (**B**) cells by RT-qPCR after the indicated period of culture. The expression of osteogenic genes *ALPL*, *COLIA1* (type I collagen), and *SPP1* (OPN) was determined in the hFOB 1.19 (**C**) and Saos-2 (**D**) cells by RT-qPCR after the indicated period of culture. The levels of calcium deposited in the extracellular matrix were determined in 24-well plates using Alizarin Red S staining. Representative micrographs (40×) of stained cultures and mean levels of ARS extracted from the hFOB 1.19 (**E**) and Saos-2 (**F**) cultures. Representative photographs of stained Saos-2 cultures (**G**). Mean ± SD from three independent experiments; * *p* < 0.05, ** *p* < 0.01, *** *p* < 0.001 in comparison to the control, one-way ANOVA with post hoc Dunnett’s test.

Moreover, we carried out an assay of osteoblast differentiation in media without additional osteogenic supplements (ascorbic acid) to evaluate whether BET alone possesses the activity to regulate osteogenesis. As shown in Figure 4A and B, BET (0.1 and 0.5 µM) increased the expression of the *RUNX2* gene in both the hFOB1.19 and Saos-2 cells. Statistically significant increases in the RUNX2 mRNA levels in the BET-treated osteoblasts were detected at day 6, 9, and 12 of osteoblast cultures. Further investigations revealed that BET also induced the expression of other osteoblast differentiation marker genes and enhanced mineralization, i.e., the final step of this process. As shown in Figure 4C,D, at a concentration of 0.1 and/or 0.5 µM, BET elevated the expression of all tested differentiation makers, except *ALPL* in the Saos-2 cells, at the indicated culture time.

Especially, the expression of the *COL1A1* gene increased in comparison with the control and other markers in both BET-treated cell lines. In the hFOB 1.19 cells, the expression of *COL1A1* was 2.9 and 4.5 times higher after 0.1 and 0.5 µM BET treatment than in the control, respectively (Figure 4C). In turn, the COL1A1 mRNA levels increased about 1.9 and 3.8 times in the 0.1 μM and 0.5 μM BET-treated Saos-2 cells, respectively (Figure 4D). Moreover, the matrix mineralization in the BET-treated osteoblasts was also enhanced, as indicated by ARS staining (Figure 4E–G). The microscopic analysis showed that the hFOB 1.19 and Saos-2 cells cultured only in the basal medium (without ascorbic acid and β-glycerophosphate as a phosphate ion source) were able to form calcified matrix at the indicated culture time points, although at minimal levels. However, the addition of BET (0.01 and/or 0.1 μM) significantly enhanced mineralization in both BET-treated osteoblast cultures, compared with the control. This was evidenced by the ARS quantitative analysis and the microscopic and/or macroscopic (Figure 4G) observation of the bone nodule morphology in the hFOB 1.19 and Soas-2 cultures.

### 2.4. BET Influences the Phosphorylation Status of JNK, ERK1/2, and mTOR Kinases

**Figure 5:**
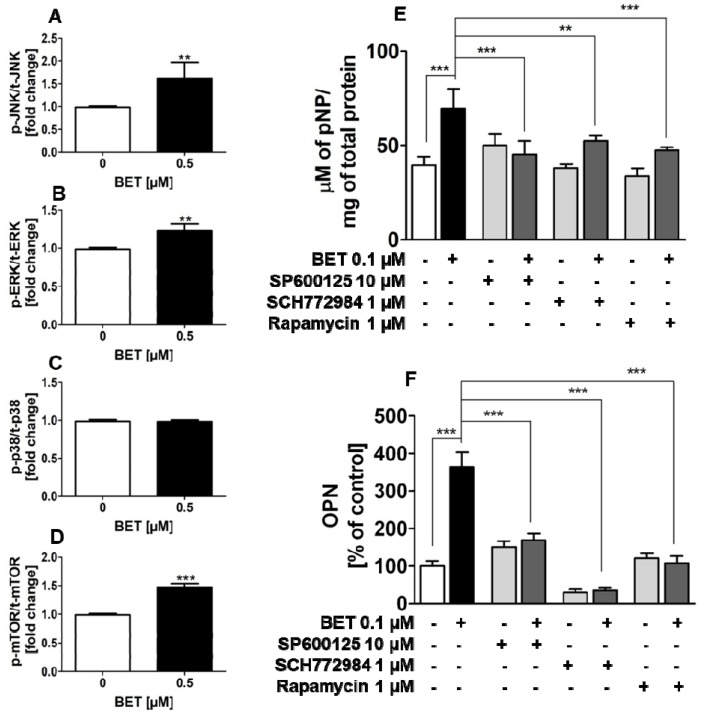
Effect of BET on the activation of JNK, ERK1/2, p38, and mTOR kinases in the hFOB 1.19 cells (**A**–**D**). Effects of JNK, ERK, and mTOR inhibitors on the levels of BET-induced ALP (**E**) and OPN (**F**). The cells were cultured in an osteoinductive medium with BET at a concentration of 0.5 µM or without the compound (control) for 6 h (JNK, ERK1/2, p38) or 24 h (mTOR). The levels of JNK (**A**), ERK1/2 (**B**), p38 (**C**), and mTOR (**D**) phosphorylation in the hFOB1.19 cells were evaluated with the ELISA method. The hFOB 1.19 cells were cultured in an osteoinductive medium with BET at a concentration of 0.1 µM or co-treated with SP600125 (5 µM), rapamycin (1 µM), or SCH772984 (1 µM). The osteoinductive medium supplemented with BET or BET plus the inhibitor was changed every three days. ALP activity was evaluated after 6 days with the colorimetric method with pNPP as a substrate for ALP. The OPN levels were measured after 9 days with the ELISA method. Mean ± SD from three independent experiments; ** *p* < 0.01, *** *p* < 0.001 in comparison to the control, one-way ANOVA with post hoc Dunnett’s test (**A**–**D**) and Tukey’s test (**E**,**F**).

To explore the involvement of MAPKs and mTOR in BET-induced osteoblast differentiation, the phosphorylation status of all three members of MAP kinases, i.e., JNK, ERK1/2, and p38, as well as mTOR kinase was examined by the quantitative ELISA method. In this part of the study, we used the hFOB 1.19 cell line as a model of normal osteoblasts. As shown in Figure 5A–D, at a concentration of 0.5 µM, BET significantly increased the phosphorylation of JNK, ERK1/1, and mTOR (ser2481) kinases, but did not activate p38 kinase in the hFOB 1.19 cells. Especially, it influenced the activation of JNK and mTOR, and only slightly increased the phosphorylation of ERK1/2 kinase.

### 2.5. BET-Induced Differentiation of hFOB 1.19 Cells Involves JNK, ERK1/2, and mTOR Activation

Since BET activated JNK, ERK1/2 and mTOR kinases in the normal human osteoblastic cell line hFOB 1.19 and all these kinases play an important role in osteoblast differentiation, further studies were undertaken to test whether the increased activity of these kinases is involved in BET-induced osteoblast differentiation. To this end, the hFOB 1.19 cells were treated with SP600125 (JNK inhibitor), SCH772984 (ERK1/2 inhibitor), or rapamycin (mTOR inhibitor) and co-treated with BET (0.1 µM). As shown in Figure 5E,F, all inhibitors significantly inhibited BET-induced ALP activity at day 6 of differentiation and OPN production at day 9 of hFOB 1.19 cell differentiation, i.e., an early and terminal marker of osteoblast differentiation, respectively. Thus, the results showed that the activation of JNK, ERK1/2, and mTOR is a critical factor in BET-induced differentiation of normal osteoblasts.

## 3. Discussion

Osteoporosis is now and will be a future health problem affecting Western countries due to the increasing longevity. Although new anti-osteoporotic agents with well-documented efficacy have been developed, there is a still need for a more effective anti-fracture strategy [36]. Therefore, new molecules that inhibit osteoclast formation and/or stimulate osteoblast differentiation and can be useful in the development of new anti-osteoporotic drugs are still being sought.

Betulin is a very interesting naturally occurring compound with a broad spectrum of biological activity [37]. Recently, it has been shown that BET possesses an ability to suppress osteoclast formation [38], which suggests that it has potential for prevention and/or therapeutic treatment of loss of bone mass. However, the osteogenic potential of BET has not been investigated so far. Moreover, only a few studies have been carried out on the osteogenic potential of some triterpenoids. For example, Lo et al. [30] showed the osteogenic potential of a betulin derivative, namely betulinic acid, in murine pre-osteoblast cell line MC3T3-E1. Moreover, Lee et al. [28] and Shim et al. [29] showed that ursolic acid and corosolic acid enhanced differentiation and mineralization of osteoblasts in the same murine osteoblast model (MC3T3-E1). Additionally, ursolic acid had bone-forming activity in vivo in a mouse calvarial bone formation model [28]. Thus, all these studies suggest that triterpenoids can stimulate osteoblast differentiation and enhance new bone formation. However, in all of these studies, only murine osteoblast models were used and the osteogenic potential of these compounds was tested in vitro in media with additional supplementation of ascorbic acid, i.e., a compound with well-established osteogenic activity [32,33].

In this study, we used two human osteoblast cell lines, i.e., a normal osteoblast cell line hFOB 1.19 and a neoplastic osteoblast-like cell line Saos-2. These commonly used models of osteoblast differentiation for in vitro research [39] were used to evaluate the effect of BET on osteoblast differentiation and mineralization in the osteogenic conditions. Moreover, the osteoinductive potential of BET in both models of osteoblasts and the underlying mechanism of its action in hFOB 1.19 cells were investigated. First, we evaluated the cytotoxic effect of BET in both cell lines. Although the results showed that BET exerted low cytotoxic activity against the hFOB 1.19 cells and higher cytotoxic activity against the Saos-2 cells, it significantly inhibited proliferation of both cell lines at such a low concentration as 1 μM. There are some reports indicating that BET exerted low cytotoxicity against human normal cells. For example, at concentrations below 10 μM, BET exerted no toxicity against human skin fibroblasts (HSF) [16]. Also, low cytotoxicity towards immortalized human epithelial cells (hTERT-RPE1 cell line) and human umbilical vein endothelial cells (HUVEC) with the IC_50_ value > 45 μM has been shown [40]. On the other hand, BET has been shown to exert a significant anti-proliferative effect against both human normal cells and neoplastic cells. For example, BET inhibited proliferation of human normal skin fibroblasts (WS1 cell line) with the IC_50_ value of 3.6 μM [41,42] and human neuroblastoma cells (SK-N-AS cell line) with the IC_50_ value of 2.5 μM [16]. Thus, the results of our study on the effect of BET on osteoblast proliferation are consistent with the results of other studies.

Given the significant anti-proliferative activity of BET towards human osteoblasts, in the studies on the osteogenic/osteoinductive potential of this compound, BET was tested only at low concentrations. Our present study showed a stimulatory effect of BET on the differentiation of osteoblasts in both the osteogenic and osteoinductive conditions.

During osteoblast differentiation in vitro, three phases of osteoblast phenotype development leading to bone formation can be distinguished: (1) cell proliferation; (2) extracellular matrix synthesis and maturation; (3) terminal differentiation with matrix mineralization. During these steps, the expression of appropriate genes/markers occurs temporally and sequentially, and the levels of expression of these genes are often used to monitor the progression of differentiation [43]. The induction of osteoblast differentiation is closely related to the positive regulation of RUNX2. Both, overexpression and/or elevated transcriptional activity of RUNX2 influence osteoblastic differentiation at the early stage [7,44]. It is detected in pre-osteoblasts and its expression is upregulated in immature osteoblasts, but downregulated in mature osteoblasts [7]. In our experiments, BET up-regulated *RUNX2* gene expression especially at day 9 and 12 of culture in both cell lines in the osteogenic and osteoinductive conditions. Consistently, BET significantly increased the production and/or mRNA expression of markers of osteoblast maturation and differentiation such as ALP, type I collagen and OPN, in both the osteogenic and osteoinductive conditions, except the ALP expression/activity in the Saos-2 cells. These results indicated indirectly that BET positively regulated RUNX2 because *ALPL*, *COL1A1* and *OPN* are target genes of this transcription factor [7,45]. However, as we did not examine the protein level of RUNX2 in the BET-treated osteoblasts, we can only suppose that BET could stimulate/induce osteoblast differentiation by increasing the level of this transcription factor or by increasing its transcriptional activity. It is well known that post-translational modifications such as phosphorylation and acetylation affect the transcriptional activity of RUNX2 [46]. For example, phosphorylation and activation of RUNX2 has been shown to be mediated by different kinases including ERK, protein kinases A and Cδ (PKA, PKCδ), Akt, homeodomain-interacting protein kinase 3 (HIPK3) or cyclin-dependent kinase-1 (CDK1). Also, ERK signaling-dependent acetylation results in stimulatory effect on RUNX2 activation and stability [46]. Given the multiple possible ways of RUNX2 regulation [46], this issue needs separate studies to determine the detailed mechanism of regulation of this transcription factor by BET in human osteoblasts.

The appearance of ALP activity is an early phenotypic marker of osteoblast maturation and synthesis of this enzyme is enhanced along the process of cell differentiation [47]. In our study, BET increased the expression of the *ALP* gene in the hFOB 1.19 cells in both the osteogenic and osteoinductive conditions, which suggests that the triterpene influenced an early stage of differentiation of these cells. In contrast, BET decreased the expression of the *ALP* gene in the Saos-2 cells at day 9 of culture in the osteoinductive conditions and ALP activity in these cells at the same time point in the osteogenic conditions. However, in contrast to hFOB 1.19 pre-osteoblasts, Saos-2 cells have a mature osteoblast phenotype with a high endogenous level of ALP activity [39]. Thus, the phenomenon observed may suggest that BET accelerated the differentiation of the Saos-2 cells.

Type I collagen is the main protein of bone matrix and acts as a scaffold for the nucleation of hydroxyapatite crystals. It is a phenotypic marker for a late stage of osteoblast differentiation. In turn, OPN is a non-collagenous bone matrix protein synthesized and secreted by osteoblastic cells in the terminal stage of differentiation [43]. In our experiments, the treatment of the hFOB1.19 and Saos-2 cells with BET increased the expression of *COL1A1* and *SPP1* (OPN) genes in both the osteogenic and osteoinductive conditions, which additionally correlated directly to the protein level of these markers in the osteogenic conditions. Thus, our results proved that BET stimulated the late stages of osteoblast differentiation as well. Moreover, the osteogenic/osteoinductive activity of BET was further proved, as the treatment of both the hFOB 1.19 and Saos-2 cells with BET enhanced the formation of mineralized nodules significantly, which is a morphological manifestation of osteogenesis.

There are reports indicating that BET has ability to interact with melanocortin receptors [48], which are also detected on osteoblasts [49]. However, BET itself did not stimulate cyclic adenosine 3′,5′-monophosphate (cAMP), i.e., second messenger, generation after melanocortin receptor ligation [48]. On the other hand, BET is a compound with high cell permeability [50], which suggests that it can directly activate different signaling pathways after entering into cells. For example, it has been shown that BET activates the signal transducer and activator of transcription 3 (STAT3) pathway [51]. For this reason, we can suppose that BET had osteogenic activity through direct activation of some kinases.

Since osteogenesis is mediated among others by JNK, ERK1/2, and p38 signaling pathways [8], we evaluated the activation of these kinases in order to elucidate the mechanism by which BET acts on osteoblasts. Considering the osteoblast differentiation, ERK has been reported to regulate the process by regulating the activity of RUNX2 [52]. It was described that numerous bone-active factors, including fibroblast growth factor (FGF), insulin-like growth factors (IGFs), or estrogen, induce ERK signaling in osteoblasts [8,53]. The p38 signaling in turn is activated by TGF-β and bone morphogenetic proteins (BMPs), and controls RUNX2 activity as well. However, recent evidence suggests that ERK and p38 may be responsible for different osteoblast responses [8]. The JNK signaling is activated by cytokines, including BMPs, and different kinds of stress [53]. This pathway is involved in osteoblast differentiation through activation of the osteogenesis-related transcription factors such as AP-1 or ATF4 [12,54]. In the present study, BET enhanced ERK1/2 and JNK phosphorylation in the hFOB 1.19 cells although it did not affect p38 activation. Moreover, pretreatment of these cells with the JNK inhibitor (SP600125) or ERK inhibitor (SCH772984) abolished the BET-induced increase in the ALP activity and OPN production. These data suggest an essential role of the JNK and ERK signaling pathways in BET-induced osteoblast differentiation.

mTOR kinase is a component of two distinct multi-protein complexes, mTORC1 and mTORC2, which can be distinguished by their essential factors such as Raptor for mTORC1 and Rictor for mTORC2 [55]. mTOR is a central coordinator of fundamental biological processes such as cell growth, cell-cycle progression, and cell differentiation. It is activated by a multitude of intracellular end extracellular signals [55]. Both mTOR complexes are activated by various growth factors including bone anabolic signals such as IGF-1, Wnt, or BMP-2 [56,57,58,59,60]. Recent studies have established that mTORC1 and mTORC2 are implicated in regulating osteoblast differentiation and function [9]. Moreover, it has been shown that rapamycin, a specific inhibitor of mTOR, significantly reduced the levels of the Runx2 protein and the expression of other osteoblast differentiation markers in differentiating osteoblasts [61]. Our present study demonstrated for the first time that BET at low concentrations induced phosphorylation of mTOR in human osteoblasts. Moreover, pretreatment of these cells with rapamycin abolished the BET-induced increase in the ALP activity and OPN production, indicating the importance of the mTOR pathway in BET-induced osteoblast differentiation.

### 4.1. Reagent

Betulin (BET) was purchased from Sigma-Aldrich Chemicals (St. Louis, MO, USA). A stock solution of BET was prepared in DMSO (Sigma) and stored at 4 °C until use. The final concentration of DMSO in all experiments was lower than 0.01%, and all treatment conditions were compared with vehicle controls. The triterpenoid stock solution was diluted in a culture medium immediately before use.

### 4.2. Cell Cultures

The human fetal osteoblastic cell line hFOB 1.19 (CRL-11372^TM^) was obtained from the American Type Culture Collection (ATCC, Manassas, VA, USA). The cells were maintained in a growth medium containing a 1:1 mixture of Dulbecco’s Modified Eagle’s Medium without phenol red and Ham’s F12 medium, supplemented with 2.5 mM of l-glutamine, 0.3 mg/mL of G418, antibiotic/antimycotic solution (all from Sigma), and 10% of fetal bovine serum, FBS (Thermo Fisher Scientific, Gibco, Waltham, MA, USA) in a humidified 5% CO_2_ atmosphere at a permissive temperature of 34 °C. The human osteoblast-like cell line of osteosarcoma Saos-2 (HTB-85^TM^) was purchased in ATCC. Saos-2 cells were maintained in McCoy’s 5A Modified Medium (Sigma) supplemented with an antibiotic/antimycotic solution and 10% FBS in a humidified 5% CO_2_ atmosphere at a temperature of 37 °C.

The modulatory influence of BET on differentiation of osteoblasts was examined in cells cultivated in osteogenic media, which consisted of the basal medium for each cell line supplemented with 1% (hFOB 1.19) or 2% (Saos-2) FBS and additionally 10 mM of β-glycerophosphate (Sigma) (hFOB 1.19) and 50 µg/mL of ascorbic phosphate-magnesium salt (Sigma) (both cell lines). The osteoinductive activity of BET was examined in cells cultivated in basal medium with 1% (hFOB1.19) or 2% (Saos-2) FBS and without any osteogenic supplements (osteoinductive medium). During the osteoblast differentiation experiments, the media were changed every three days and the cells were cultured for up to 18 days.

### 4.3. Cytotoxicity Assay

The cytotoxicity of BET towards osteoblasts was estimated by measurement of lactate dehydrogenase (LDH) activity (Tox-7, Sigma). The cells were seeded in 96-well microplates at a density of 3 × 10^4^ cells/well (hFOB 1.19) or 5 × 10^4^ cells/well (Saos-2) in 100 μL of a complete growth medium. The next day, the medium was changed to a new one containing 1% FBS and the cells were exposed to various concentrations of BET (0.1–25 µM). After 24 h of incubation, LDH activity was examined in culture supernatants according to the manufacturer’s instruction. The absorbance measurement was performed at a wavelength of 450 nm using an E-max Microplate Reader (Molecular Devices Corporation, Menlo Park, CA, USA).

### 4.4. Cell Proliferation Assay

The cells were seeded into 96-well plates at a density of 3 × 10^3^ cells/well (hFOB 1.19) or 5 × 10^3^ cells/well (Saos-2) in 100 μL of a complete growth medium. After 24 h, the culture medium was removed and the cells were exposed to BET (0.01–25 µM). Cell proliferation was measured after 96 h of incubation by the MTT assay. The cells were incubated for 3 h with 20 microliters of 5 mg/mL 3-(4,5-dimethylthiazol-2-yl)-2,5-diphenyltetrazolium bromide solution (MTT, Sigma). Next, formazan crystals were solubilized overnight in SDS buffer (10 % SDS in 0.01 N HCl) and absorbance of the product was measured at a wavelength of 570 nm using the E-max Microplate Reader (Molecular Devices Corporation, Menlo Park, CA, USA).

The expression of *ALPL*, *COL1A1*, *SPP1*, *Runx2*, and β-actin (*ACTB*) genes were analyzed by quantitative real-time PCR (RT-qPCR). After cell harvesting, total cellular RNA from each sample was isolated using the QIAamp^®^ RNA Blood Mini Kit (QIAGEN^®^ GmbH, Hilden, Germany) according to the manufacturer’s protocol. cDNA was synthesized by reverse transcription of 1 µg of total RNA for 30 min at 42 °C using a QuantiTect^®^Reverse Transcription Kit (QIAGEN^®^) according to the manufacturer’s protocol. RT-qPCR was performed using a TaqMan Gene Expression Assay probe/primer specific for each analyzed marker (*ALPL* – Hs01029144, *COL1A1* – Hs00164004, *SPP1* – Hs00959010, *Runx2* - Hs00231692) and internal control β-actin (human ACTB Endogenous Control VIC/MGB Probe, Applied Biosystems, Carlsbad, CA, USA). The real-time PCR was performed using 1 µL of cDNA template of each sample and 2.5 µL of the specific primer in 50 µL of the reaction mixture. The amplification was performed on a CFX96™ Real-Time PCR Detection System C1000 Touch™ (Bio-Rad Laboratories, Hercules, CA, USA) using the following cycle parameters: 95 °C for 10 min followed by 40 cycles (or 60 cycles in the case of Runx2) at 95 °C for 15 s and 60 °C for 1 min. All reactions were run in triplicate, and data were analyzed using the threshold cycle (CT) and the 2^-∆CT^ method. The mRNA level in the control (untreated cells; 0 µM) was considered as 1. The results of gene expression were presented as a fold change in comparison with the control.

### 4.5. Alkaline Phosphatase Activity Assay

The ALP activity was assessed using the colorimetric method. To this end, the cells were plated on 96-well microplates (5 × 10^3^ cells/well) in the growth medium and cultivated for 2-3 days to obtain a confluent monolayer. Then, the cells were grown in the osteogenic medium without or with BET (0.01 and 0.1 µM). In some experiments, the hFOB 1.19 cells were exposed to BET (0.1 µM) without or in combination with SCH772984 (an ERK1/2 inhibitor, 1 μM, Selleck Chemicals LLC, Houston, TX, USA), SP600125 (a JNK inhibitor, 5 μM, Sigma), or rapamycin (an mTOR inhibitor, 1 µM, Sigma) in osteoinductive conditions. ALP activity was assayed as previously described [58]. The absorbance was determined at a wavelength of 405 nm using an E-max Microplate Reader (Molecular Devices Corporation, Menlo Park, CA, USA). The total protein concentration in the cell lysates was determined by incubation with a bicinchoninic acid protein assay reagent (Pierce^®^ BCA Protein Assay Kit; Thermo Fisher Scientific, Rockford, IL, USA). ALP activity was calibrated with a p-nitrophenol (pNP) standard curve and calculated as µM of pNP/mg of total protein concentration.

### 4.6. Western Blot Analysis of Type I Collagen Level

The collagen type 1A1 (Col1A1) level was determined by western blot analysis as previously described [62]. Briefly, the cells were seeded into 25 cm^2^ plastic flasks (Nunc, Roskilde, Denmark). After 2–3 days of incubation, the growth medium was removed and the osteogenic medium without or with BET (0.01 and 0.1 µM) was added. After an appropriate incubation time, cells were harvested, lysed in RIPA buffer (Sigma) supplemented with protease and phosphatase inhibitor cocktail (Sigma), and centrifuged (14,000 rpm for 10 min at 4 °C). The total protein concentrations were determined using a Pierce BCA Protein Assay Kit (Thermo Fisher Scientific) according to the manufacturer’s instructions. Primary rabbit polyclonal antibodies against collagen α1 type I (COL1A1) of human origin (1:500; sc8784-R) were purchased from Santa Cruz Biotechnology Inc. (Dallas, TX, USA). The primary antibodies were detected by horseradish peroxidase (HRP)-conjugated goat anti-rabbit secondary antibodies (Amersham^TM^ GE Healthcare, Buckinghamshire, UK). The enhanced chemiluminescence method (ECLTM Western Blotting Analysis System, Amersham Bioscience) was used to detect protein bands according to the manufacturer’s protocol. After incubation with the substrate, membranes were visualized and analyzed by means of Molecular Imager^®^ChemiDoc^TM^ XRS+ (Bio-Rad Laboratories, Hercules, CA, USA) equipped with Image Lab^TM^ Version 3.0 Software for measurement of protein band intensity. The COL1A1 bands were determined on the basis of molecular mass using Precision Plus Protein^TM^ Standards (Bio-Rad Laboratories, Hercules, CA, USA). The blots used for collagen detection were stripped and reprobed with anti β-actin antibodies (1:500, AC-74, Sigma) used as a load control. The COL1A1 band densities were normalized to that of β-actin. The changes in the COL1A1 level were expressed as a % of the control level (100%).

### 4.7. Assay of Osteopontin Production

The concentrations of OPN in the cell culture media were estimated by the ELISA method. Cells were seeded into 24-well plates in the growth medium. After 24 h, the culture medium was removed and the osteogenic medium without or with BET (0.01 and 0.1 µM) was added. In some experiments, hFOB 1.19 cells were exposed to BET (0.1 µM) without or in combination with SCH772984 (an ERK inhibitor, 1μM), SP600125 (a JNK1/2 inhibitor, 5 μM), or rapamycin (1 µM) in osteoinductive conditions. After the indicated time of incubation, the cell culture media were collected, centrifuged, and stored at −80 °C. The OPN level was measured using a RayBio^®^ Human Osteopontin ELISA kit (RayBiotech, Inc., Atlanta, GA, USA) according the manufacturer’s protocol. The OPN concentration in control samples (without BET) was considered as 100%. The OPN level in samples collected from the BET and/or inhibitors-treated cell cultures were expressed as a % of control.

### 4.8. Alizarin Red S Staining

The degree of the extracellular matrix mineralization was determined using Alizarin Red S (ARS, Sigma) staining of cultured osteoblasts to detect bone nodules (calcium precipitates) as previously described [63]. The ARS-stained cell cultures were examined under a light microscope. Finally, the incorporated ARS dye was extracted from the cell cultures with 10% cetylpiridinium chloride in 10 mM sodium phosphate (pH = 7) for 1 h at 37 °C. The extracted stain was transferred to a 96-well plate and the absorbance at 562 nm was measured using an E-max Microplate Reader. The level of ARS stain extracted from the control cell cultures was considered as 100%. The calcium deposition level in the BET-treated cell cultures was expressed as % of control.

### 4.9. ELISA Assay of MAP Kinases and mTOR Activation

The quantification of the intracellular levels of total and phosphorylated JNK, ERK1/2, p38, and mTOR kinases in the BET-treated hFOB 1.19 cells was carried out using the following PathScan^®^ ELISA kits: phospho-SAPK/JNK (Thr183/Tyr185), total SAPK/JNK, phospho-p44/42 MAPK (Thr202/Tyr204), total p44/42 (ERK 1/2), phospho-p38 MAPK (Thr180/Tyr182), phospho-mTOR (Ser2448) and total mTOR Sandwich ELISA Kit (Cell Signaling Technology, Danvers, MA, USA), and p38 MAPK alpha ELISA Kit (Abcam, Cambridge, UK), according to the manufacturer’s instructions. Briefly, hFOB 1.19 cells were seeded into 10-cm diameter plastic plates and cultured in the growth medium until confluency. Then, the medium was removed and the osteoinductive medium supplemented (or not) with BET (0.5 µM) was added. After 6 or 24 h of exposure, the media were removed and the cells were lysed in lysis buffer supplemented with PMSF (Sigma) and protease and phosphatase inhibitor cocktail (Sigma) according to the manufacturer’s protocol. The cell lysates were centrifuged at 14,000 rpm for 5 min at 4 °C and stored at −80 °C until analysis. Before the assay, the total protein concentrations in the cell lysates were determined with a Pierce BCA Protein Assay Kit (Thermo Fisher Scientific), and samples containing equal amounts of total proteins per 100 µL of sample diluent were subjected to ELISA.

### 4.10. Statistical Analyses

Each experiment was repeated at least three times. Statistical analyses were performed using GraphPAD Prism v5 (GraphPAD Software Inc., San Diego, CA, USA). The data were analyzed by one-way ANOVA followed by Dunnett’s or Tukey’s tests for multiple comparisons between pairs. The *p* values < 0.05 were considered statistically significant.

## 5. Conclusions

In summary, the present study demonstrated that betulin promoted osteogenesis in vitro in human osteoblasts models in osteogenic conditions. Moreover, our studies suggest that BET can induce osteoblast differentiation via JNK, ERK1/2 and mTOR kinase-dependent signaling pathways. Noteworthy, BET exerted a pro-osteogenic effect at the low, non-toxic concentrations. Given the satisfactory bioavailability of BET at the diverse routes of administration [64,65], we can conclude that this compound has the potential to be used as a medicine or supplement in treatment of bone osteoporotic lesions, especially since the recent studies [38] indicated the anti-osteoclastic activity of BET. However, further in vivo studies in animal models are necessary to confirm the potential of BET to enhance osteogenesis.
